# Comparison of the I-Gel and the Laryngeal Mask Airway Proseal during General Anesthesia: A Systematic Review and Meta-Analysis

**DOI:** 10.1371/journal.pone.0119469

**Published:** 2015-03-26

**Authors:** Sun Kyung Park, Geun Joo Choi, Yun Suk Choi, Eun Jin Ahn, Hyun Kang

**Affiliations:** 1 Department of Anesthesiology and Pain Medicine, College of Medicine, Jeju National University, JeJu, Korea; 2 Department of Anesthesiology and Pain Medicine, Chung-Ang University College of Medicine, Seoul, Korea; 3 Department of Anesthesiology and Pain Medicine, SungKyunKwan University Hospital, College of Medicine, Seoul, Korea; University of Colorado, UNITED STATES

## Abstract

**Objectives:**

Conflicting results have been reported for the i-gel and the laryngeal mask airway proseal (LMA-P) during general anesthesia. The objective of the current investigation was to compare the efficacy and safety of the i-gel vs. the LMA-P during general anesthesia.

**Methods:**

Two authors performed searches of MEDLINE, EMBASE, CENTRAL, and Google Scholar to identify randomized clinical trials that compared the LMA-P with the i-gel during general anesthesia. A meta -analysis was performed using both random and fixed-effect models. Publication bias was evaluated using Begg's funnel plot and Egger's linear regression test.

**Results:**

Twelve randomized clinical trials met the eligibility criteria. There were no significant differences in insertion success rate at the first attempt (risk ratio [RR] 1.01, 95% confidence interval [CI] 0.97, 1.06), ease of insertion (RR 1.14, 95% CI 0.93, 1.39), oropharyngeal leak pressure (OLP) (MD -1.98, 95% CI -5.41, 1.45), quality of fiberoptic view (RR 1.00, 95% CI 0.91, 1.10) and success rate of gastric tube insertion (RR 1.07, 95% CI 0.98, 1.18) between the i-gel and the LMA-P, respectively. The i-gel had a shorter insertion time than the LMA-P (MD -3.99, 95% CI -7.13, -0.84) and a lower incidence of blood staining on the device (RR 0.26, 95% CI 0.14, 0.49), sore throat (RR 0.28, 95% CI 0.15, 0.50) and dysphagia (RR 0.27, 95% CI 0.10, 0.74).

**Conclusions:**

Both devices were comparable in ease of insertion to insert and both had sufficient OLP to provide a reliable airway. Only a few minor complications were reported. The i-gel was found to have fewer complications (blood staining, sore throat, dysphagia) than the LMA-P and offers certain advantages over the LMA-P in adults under general anesthesia.

## Introduction

Supraglottic airway devices (SGAs) have been shown to be suitable for use in routine anesthesia and emergency airway procedures [1]. The i-gel airway (Intersurgical Ltd, Workingham., Berkshire, United Kingdom) and the ProSeal LMA (LMA-P) are second generation SGAs that were introduced in 2007 and 2000, respectively [2]. The i-gel is a single use SGA composed of a soft, gel-like, non-inflatable cuff made from a thermoplastic elastomer. It has a widened, flattened stem with a rigid bite block that acts as a buccal stabilizer to reduce axial rotation and mal-positioning, and a port for gastric tube insertion. It is a latex free device that does not require digital insertion into patient’s mouths, and it is less expensive than other SGAs [3,4]. The LMA-P is an inflatable device with a drain tube (DT), parallel to the ventilation tube, passes through the bowl of the mask [4]. The posterior inflatable cuff and the increased bulk of the LMA-P substantially increase the pharyngeal seal [5]. Because of the larger bulk of the LMA-P tip and the absence of a back-plate on the device, poor insertion technique results in posterior folding over of the device. There are 3 insertion techniques for the LMA-P (standard, introducer, and bougie-guided) [6,7].

Both devices provide a higher oropharyngeal leak pressure (OLP) than the classic LMA (CLMA) [5,8,9], incorporate an additional drain tube and are designed for use in spontaneous as well as positive pressure ventilation [10]. There has been significant interest in SGAs that incorporate a DT, which enables identification of mask misplacement and can also reduce the risk of gastric content aspiration [7]. Several studies have compared the safety and efficacy of the i-gel and the LMA-P, but the results have been inconsistent. [4,10–15]

To our knowledge, no previous systematic review comparing the LMA-P and i-gel has been published. To address this deficiency, we conducted a systematic review and meta-analysis comparing the two devices.

The objective of this systematic review was to compare the clinical performance of the LMA-P and i-gel by evaluating the available literature. Clinical performance including the insertion success rate on the first attempt, ease of insertion, insertion time, OLP, and the quality of the fiberoptic view were the primary focus. The frequency of complications, when reported, was also assessed, and the limitations of the data were reviewed.

## Methods

The present systematic review was registered in PROSPERO (CRD42014007422)

### Literature search

Following the protocol recommended by the Cochrane Collaboration, we performed a systematic the literature search for studies that compared the i-gel with the LMA-P in adult patients undergoing general anesthesia [16]. A Search was performed in MEDLINE, EMBASE, CENTRAL, and Google Scholar in January 2014, and updated in September 2014.

The terms used to search MEDLINE and EMBASE are presented in the S1 Appendix. We also searched the bibliographies of relevant articles to identify additional studies.

### Study selection

The studies included in our analysis were peer-reviewed, randomized controlled trials that compared i-gel with LMA-P in adult patients undergoing general anesthesia, and reported clinical performance and/or complications. All studies that met these criteria were included regardless of publication language. Review articles, case reports, case-series, letters to the editor, commentaries, proceedings, laboratory science studies, comparative studies using manikins, and any other non-relevant studies were excluded. Two authors (G.J.C. and S.K.P) independently selected eligible studies, and then discussed any differences of opinion in order to arrive at a consensus as to whether a study should be included or excluded. Disagreements over inclusion were settled after discussion with the senior author (H.K.).

### Data extraction

Two authors (G.J.C. and S.K.P.) independently recorded the following data using standardized forms: the first author’s name; year of publication; number of patients; demographic data (age and weight); size of the devices used; type of surgery; use of neuromuscular blocking drugs; insertion time; insertion success rate at the first attempt; ease of insertion; OLP; quality of fibreoptic view; success rate of gastric tube insertion; complications during anesthesia (including coughing, hypoxia, bronchospasm, gastric insufflation, mouth or tongue injury, regurgitation and blood staining on the device); and complications after anesthesia (including, hoarseness, sore throat and dysphagia). We also recorded the conflict of interest information from each study.

When studies compared multiple airway devices, only the data for the i-gel and LMA-P were evaluated. For studies with insufficient or missing data, we attempted to contact the authors. If this was unsuccessful, we extrapolated the data from the text or tables, or used other reported parameters to calculate the target information.

### Risk of bias assessment

The quality of the studies was independently assessed by 2 authors (G.J.C. and S.K.P) using the Review Manager (version 5.1, The Cochrane Collaboration, Oxford, UK) ‘risk of bias’ tool. Quality was evaluated using the following 7 potential sources of bias: random sequence generation, allocation concealment, blinding of the participants, blinding of outcome assessment, incomplete outcome data, and selective reporting. The methodology for each trial was graded as “high,” “low,” or “unclear,” to reflect either a high, low or uncertain risk of bias, respectively.

### Statistical analysis

The pooled RR or MD and 95% CIs were calculated for each outcome using Review Manager (version 5.1, The Cochrane Collaboration, Oxford, UK), and Comprehensive Meta- Analysis (version 2.0, Biostat, Inc., Englewood, USA) software. We used the chi-squared test for homogeneity and the I^2^ test for heterogeneity. A level of 10% significance (P < 0.1) for the chi-squared statistic or an I^2^ greater than 50% was considered to indicate considerable heterogeneity. The Mantel–Haenszel random-effect model was used for these studies. The Mantel–Haenszel fixed model was used for studies that did not demonstrate significant heterogeneity [16,17]. We performed subgroup analyses based on the use of a neuromuscular blocking drug and the type of surgery (laparoscopic versus non-laparoscopic). To assess the heterogeneity of outcomes, we performed sensitivity analysis to evaluate the influence of a single study on the overall effect estimated by excluding one study at a time.

We estimated publication bias using Begg's funnel plot and Egger's linear regression test. If the funnel plot was visually asymmetrical *or* the *P* value was found to be <0.1 using the Egger's linear regression test, the presence of a possible publication bias was identified, and trim and fill analysis was performed [18].

## Results

The results of this study are described according to recommendations given in the Preferred Reporting Items for Systematic Reviews and Meta-Analysis (PRISMA) statement [19].

### Study search

**Fig 1:**
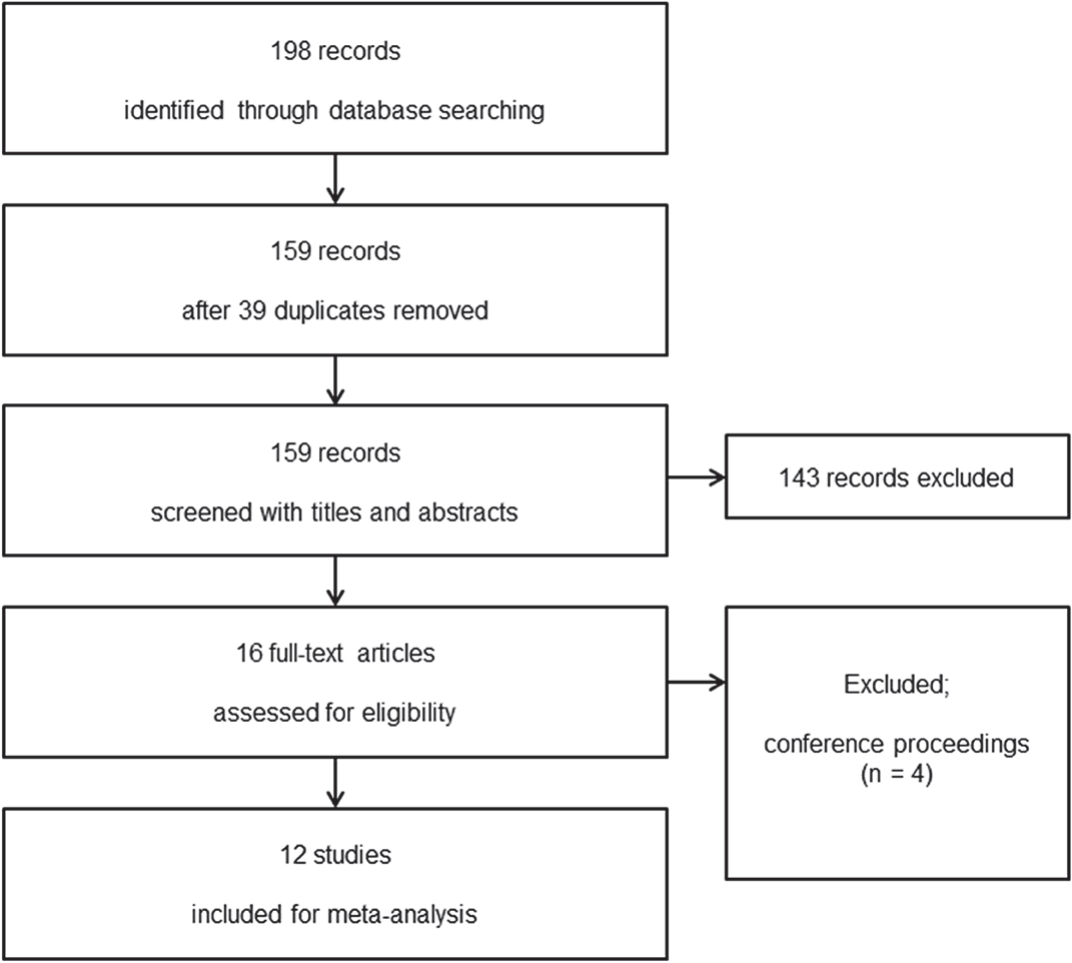
Flow diagram showing the number of abstracts and articles identified and evaluated during the review

The database search identified, 159 potentially relevant studies after the exclusion of 39 duplicates. Fourteen records were identified by screening titles and abstracts, of which 4 were found to be conference proceedings and were excluded. A total of 12 studies were included in the present systematic review (Fig. 1).

### Study characteristics and data extraction

The study characteristics are summarized in Table 1. The sample size used in the studies ranged from 30 to 152 with a total of 927 patients. The publication languages were Chinese (n = 1) [20], Japanese (n = 1) [21], and English (n = 10) [2,4,10–15,22,23]. All patients underwent a form of elective surgery. Procedures included peripheral or superficial surgery in the supine position [15], gynecological or orthopedic surgery in the supine position [13], laparoscopic cholecystectomy [10], laparoscopic gynecological surgery [14,20], surgery in the supine position [12,21,23], hernioplasty, laparoscopic cholecystectomy, tibial plating, humeral plating and skin grafting [4], lower extremity orthopedic surgery [11], and surgery under general anesthesia [2,22].

**Table 1:** Study characteristics. Values are numbers, mean ± SD, or median (range) or absolute number.

First author, year, reference	Group	N	Age	Weight	OLP* measurement method	Type of surgery	Use of NMBD†
Gasteiger, 2010 [13]	i-gel	76	40 ± 13	65 ± 11	manometric	elective gynaecological or orthopaedic surgery in supine position	No
	PLMA‡	76	41 ± 12	65 ± 11			
Van Zundert, 2012 [15]	i-gel	50	41 ± 14	71 ± 14	manometric	elective peripheral or superficial surgery in supine position (≥30 min)	No
	PLMA	50	43 ± 15	73 ± 13			
	SLMA	50	41 ± 15	74 ± 14			
Sharma, 2010 [10]	i-gel	30	42.10 ± 11.40	57.1 ± 8.48	audible	elective laparoscopic cholecystectomy	Yes
	PLMA	30	35.43 ± 11.10	58.15 ± 11.25			
Chauhan, 2013 [12]	i-gel	40	32.13 ± 11.69	57.1±8.48	audible	elective surgery in the supine position	Yes
	PLMA	40	32.43 ± 7.27	58.15 ± 11.25			
Singh, 2009 [4]	i-gel	30	38.31 ± 12.24	60.24 ± 10.89	audible	elective hernioplasty, laparoscopic cholecystectomy, tibial plating, humerus plating, skin grafting	Yes
	PLMA	30	39.86 ± 13.08	61.27 ± 11.85			
Jeon, 2012 [14]	i-gel	15	43 (36.5–44.0)	59.2 ± 4.4	manometric	laparoscopic gynecological operation	Yes
	PLMA	15	45 (39.7–47.8)	62.4 ± 6.6			
Shin, 2010 [11]	i-gel	64	42 ± 16	64 ± 17	manometric	lower-extremity orthopaedic surgery	Yes
	PLMA	53	44 ± 15	66 ± 12			
	CLMA§	50	48±13	64 ± 12			
Hayashi, 2013 [21]	i-gel	50	55.4 ± 18.6	61.2 ± 11.9	manometric	elective surgery in supine position	No
	PLMA	50	60.0 ± 16.8	58.4 ± 10.3			
Trivedi, 2011 [2]	i-gel	30	28.13 ± 10.01	47.9 ± 7.67	not reported	surgery under general anesthesia (≤2hours)	Yes
	PLMA	30	31.16 ± 11.16	51.4 ± 6.07			
Shi, 2013 [20]	i-gel	30	45 ± 11	62 ± 10	not reported	laparoscopic gynecological surgery	Yes
	PLMA	30	40 ± 10	61 ± 8			
Kini, 2014 [22]	i-gel	24	36.58±10.12	23.07±3.11 (BMI)	audible	elective short procedures requiring general anesthesia	No
	PLMA	24	33.63±10.60	22.35±3.33 (BMI)			
Das, 2014 [23]	i-gel	30	23.6±3	50.33±3.09	not reported	elective sugeries for < 1 hour duration in supine position	Yes
	PLMA	30	22.57±2.64	50.37±2.74			

* OLP: oropharyngeal leak pressure;

†NMBD: neuromuscular blocking drug;

‡PLMA: proseal LMA;

§CLMA: classic LMA.

The i-gel was compared with the LMA-P in 9 studies [2,4,10,12–14,21–23]. The i-gel was compared with the CLMA in addition to the LMA-P in 1 study [11]. Two studies compared the i-gel with the LMA-P and the Supreme LMA (SLMA) [15,20]. In 8 studies, induction of anesthesia was performed using a neuromuscular blocking drug [2,4,10–12,14,20,23]; 4 studies did not use neuromuscular blocking drugs [13,15,21,22]. One author, who participated in 2 of the studies [13,15], worked as a consultant for the Laryngeal Mask Company.

### Risk of bias

Nine studies [2,10–14,20,21,23] described the methods used for random sequence generation, and two studies [13,15] performed allocation concealment. Five studies reported blinding of outcome assessment [10,12,13,15,22].

### Publication bias

There was no significant publication bias found on the funnel plots. A funnel plot was used for every comparison, all of which exhibited symmetrical appearances. Egger’s linear regression method did not indicate any publication bias for the following outcomes: OLP (P = 0.782), insertion time (P = 0.373), success rate on the first attempt (P = 0.267), fiberoptic view (P = 0.775), ease of insertion (P = 0.285), success rate of gastric tube insertion (P = 0.301), sore throat (P = 0.868), or dysphagia (P = 0.786). The P values of Egger’s test for the blood staining on the devices were less than 0.1 (P = 0.006). Thus, we performed trim and fill analysis on the consumption of the presence of publication bias, but there was no change in the significance of the results (95% CI 0.28 to 0.96).

### Results of the meta-analysis

#### 1. Insertion success rate at the first attempt, ease of insertion and insertion time

In all studies, the size of the device was selected according to the manufacturer’s recommendations using the patient’s weight and the devices were inserted by experienced anesthesiologists. Eleven studies [2,4,10,11,13–15,20–23] investigated the insertion success rate at the first attempt, and no significant difference between the 2 devices (RR 1.02, 95% CI 0.97 to 1.07, P_Chi_^2^ = 0.33, I^2^ = 12%).

Four studies [4,10,12,15] measured the ease of insertion using either a 2- or 4- point scale, that was scored by an independent observer or by the clinician performing the procedure. Two studies [12,15] used the following scale: 3, insertion at the first attempt without resistance; 2, insertion at the first attempt but with some resistance; 1, insertion at the second attempt; 0, insertion failed on both attempts. Three studies [4,10,23] characterized the ease of insertion as either easy or difficult; 1 of these [4] characterized “easy” insertion as no resistance to insertion into the pharynx at the first attempt and “difficult” insertion as resistance to insertion on more than 1 attempt, while the other [10] did not provide any details. Because of the differences in the grading scale definitions, only data with a grade of “3” or “easy,” indicating an insertion with no resistance were used for the meta-analysis. There was marginal significant difference in ease of insertion between the i-gel and the LMA-P (RR 1.11, 95% CI 1.00 to 1.24, P_Chi_^2^ = 0.16, I^2^ = 42%). Sensitivity analysis was performed using a different insertion technique (guided laryngoscopy) in all but 1 trial [15]. The i-gel showed a higher frequency of insertion without resistance compared to the LMA-P (RR 1.19, 95% CI 1.04 to 1.35, P_Chi_^2^ = 0.47, I^2^ = 0%).

**Fig 2:**
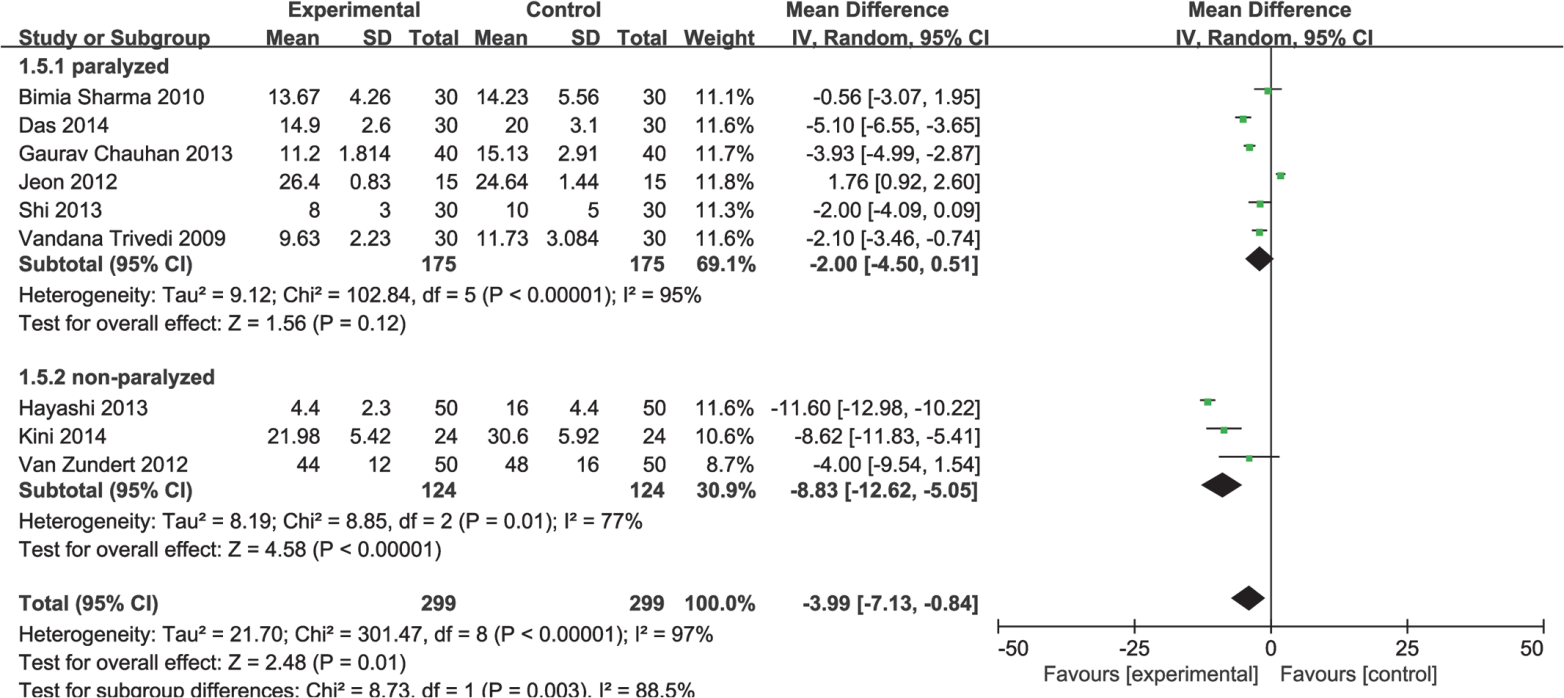
Forest plot showing insertion time: i-gel versus LMA-P. Subgroup analysis according to using of neuromuscular blocking agents (paralyzed vs. non-paralyzed).

Insertion time was measured in 9 studies [2,10,12,14,15,20–23]. It was shorter in the i-gel than in the LMA-P group (MD −3.99s, 95% CI −7.13 to −0.84, P_chi_^2^ < 0.00001, I^2^ = 97%). The definition of insertion time varied between studies. Three studies [10,20,22] did not define insertion time. Six studies [2,12,14,15,21,23] defined the insertion time as the time that elapsed between the anesthesiologist first picking up the and achievement of sufficient ventilation. Subgroup analysis was performed for studies [2,12,14,15,21] with the same measurement range for insertion time (MD −4.45 s, 95% CI −10.75 to 1.86, P_Chi_^2^ < 0.00001, I^2^ = 98%), but the result was not modified and heterogeneity was still significant. Subgroup analysis was also performed to assess the effect of using neuromuscular blocking drugs. Three trials [15,21,22] did not use a neuromuscular blocking drug during induction of anesthesia. Insertion time was significantly different in 3 studies [15,21,22] which did not use neuromuscular blocking drugs (MD −8.83s, 95% CI −12.62 to −5.05, P_chi_^2^ = 0.01, I^2^ = 77%), whereas no significant difference was found in 6 studies [2,10,12,14,20,23] that did use a neuromuscular blocking drug (MD −2.00s, 95% CI −4.50 to 0.51, P_chi_^2^ < 0.00001, I^2^ = 95%) (Fig. 2).

#### 2. OLP, fiberoptic view and rate of successful gastric tube insertion

Six studies [11,13–15,20,21] measured OLP; 5 of these used the pressure equilibrium method (manometric stability technique), while the other study did not specify methodology details [2].

**Fig 3:**
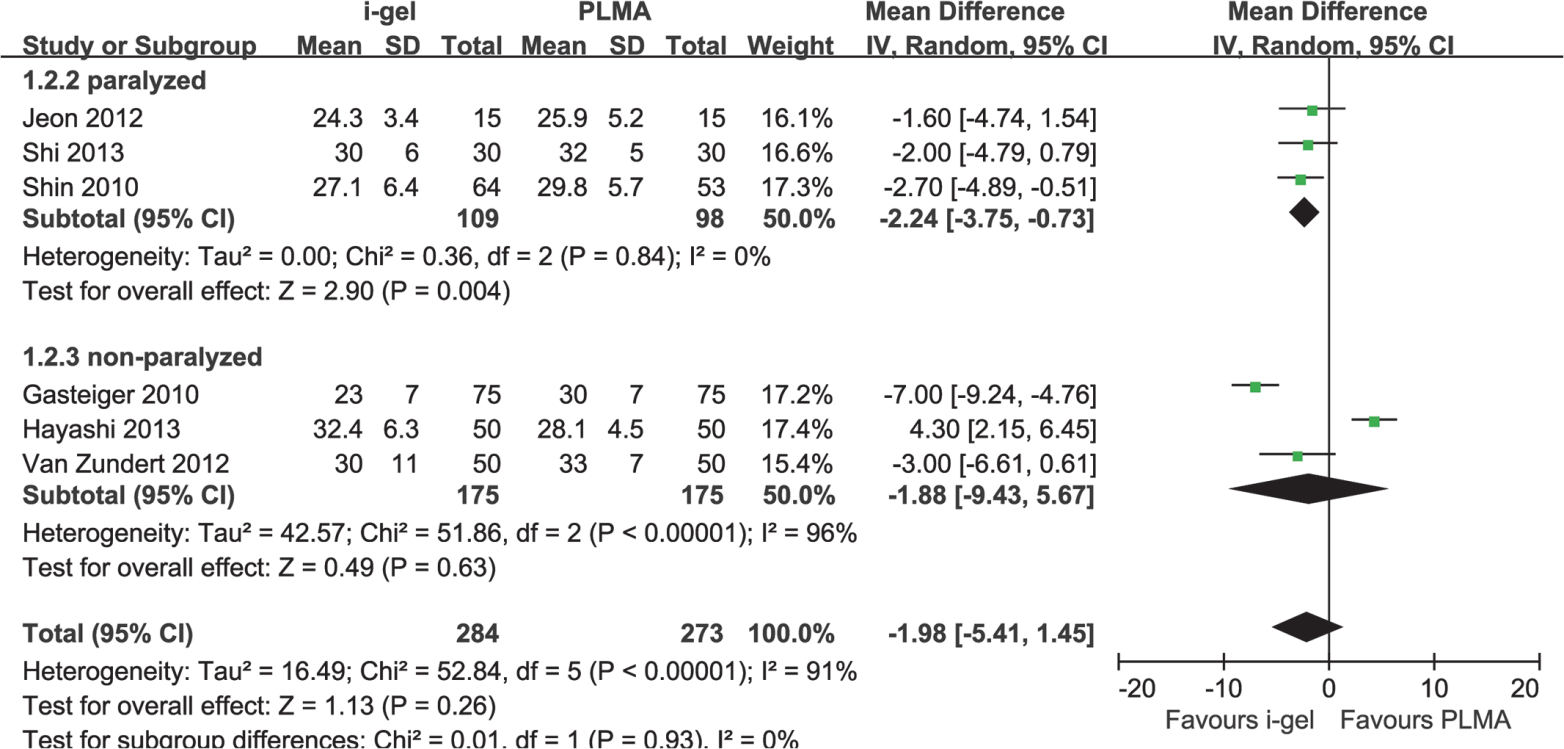
Forest plot showing oropharyngeal leak pressure: i-gel versus LMA-P. Subgroup analysis according to overall and using of neuromuscular blocking agents (paralyzed vs. non-paralyzed)

The combined OLP results showed no statistically significant difference between i-gel and LMA-P and substantial heterogeneity (MD −1.98 cmH_2_O, 95% CI −5.41 to 1.45, P_Chi_^2^ < 0.00001, I^2^ = 91%). Therefore, we performed subgroup analysis to assess the impact of neuromuscular blocking drugs. Three trials [11,14,20] that used neuromuscular blocking drugs were included, and the combined OLP results were higher for LMA-P than for i-gel (MD −2.24 cmH_2_O, 95% CI −3.75 to −0.73, P_Chi_^2^ = 0.84, I^2^ = 0%). When neuromuscular blocking drugs were nor used, the combined OLP result [13,15,21] was not statistically significant between 2 groups (MD −1.88 cmH_2_O, 95% CI −9.43 to 5.67, P_Chi_^2^ < 0.00001, I^2^ = 96%) (Fig. 3).

Subgroup analysis of the operation type (laparoscopic [14,20] versus non-laparoscopic surgery [11,13,15,21]), showed no significant difference between the 2 groups (laparoscopic: MD −1.82 cmH_2_O, 95% CI −3.91 to 0.27, P_Chi_^2^ = 0.85, I^2^ = 0% versus non laparoscopic: MD −2.08 cmH_2_O, 95% CI −7.20 to 3.05, P_Chi_^2^ < 0.00001, I^2^ = 91%).

Three studies [10,12,15] evaluated the quality of the fiberoptic view using different scales. One study [15] used the following scale: 4, only vocal cord seen; 3, vocal cords and epiglottis seen; 2, only epiglottis seen; 1, epiglottis not seen. In this study, fiberoptic views graded 3 or 4 were considered as good. Another study [12] used the following scale: 1, clear view of vocal cords; 2, only arytenoid cartilage visible; 3, only epiglottis visible; 4, no laryngeal structures visible. To simplify the analysis, fiberoptic views graded 1 or 2 were regarded as good. A third study [10] used the following scale: 4, vocal cords only; 3, vocal cords plus posterior epiglottis; 2, vocal cords plus anterior epiglottis; 1, vocal cords not seen. We classified fiberoptic views graded 3 or 4 as good. The meta-analysis showed no significance in the incidence of good fiberoptic views for i-gel compared to LMA-P (RR 1.00, 95% CI 0.91 to 1.10, P_Chi_^2^ = 0.07, I^2^ = 62%).

Six studies [4,10,12,20–22] assessed the rate of successful gastric tube insertion, which did not differ significantly between the 2 device groups (RR 1.06, 95% CI 0.98 to 1.14, P_Chi_^2^ = 0.007, I^2^ = 69%). Two studies [10,20] reported no instances of failed gastric tube insertion; 1 study [12] reported insertion failures in 2 patients from the LMA-P group; 1 study [21] reported insertion failures in 3 patients from the LMA-P group; and 1 study [4] reported insertion failures in 4 patients from the LMA-P group. No failed gastric tube insertions were reported for the i-gel group.

#### 3. Complications during and after anesthesia

The incidence of complications during anesthesia was evaluated: bronchospasm was reported in 3 studies [4,10,11]; hypoxia in 2 study [11,23]; gastric insufflations in 1 study [11]; aspiration in 3 studies [4,10,11]; regurgitation in 5 studies [4,10–12,20]; and blood staining in 9 studies [4,10–12,15,20–23].

**Fig 4:**
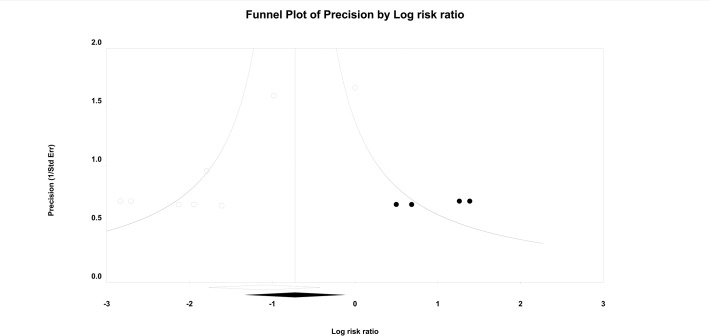
Funnel showing the incidence of blood staining on the devices: i-gel versus LMA-P. White circles: comparisons included. Black circles: inputted comparisons using the trim-and-fill method. White diamond: pooled observed log risk ratio. Black diamond: pooled inputted log risk ratio.

The incidence of complications following anesthesia was also evaluated: sore throat was reported in 10 studies [2,4,10–12,15,20–23]; dysphagia in 3 studies [12,15,20]; mouth, lip and tongue injury in 3 studies [4,11,15]; and dysphonia in 5 studies [4,12,15,20,21]. The i-gel had a significantly lower incidence of blood staining, sore throat, and dysphagia than the LMA-P (blood staining: RR 0.26, 95% CI 0.14 to 0.48, P_Chi_^2^ = 0.33, I^2^ = 13%; sore throat: RR 0.31, 95% CI 0.18 to 0.54, P_Chi_^2^ = 0.10, I^2^ = 42%; dysphagia: RR 0.27 95% CI 0.10 to 0.74, P_Chi_^2^ = 0.29, I^2^ = 20%) (Fig. 4).

There were no occurrences of aspiration, bronchospasm, hypoxia, or gastric insufflation reported in any of the studies. Complications that could be directly related to the use of the 2 devices were reported in a few cases: mouth, lip and tongue injury were reported in 1 case that used the i-gel and in 5 cases that used the LMA-P. The LMA-P was associated with 3 cases of regurgitation.

## Discussion

There was a high rate of insertion success on the first attempt for both devices reported in all the studies. This represents a clear advantage of both the i-gel and LMA-P as tools for airway management during general anesthesia. Both devices had a similar ease of insertion and produced a sufficient OLP to provide a reliable airway. Complications associated with the use of these devices were uncommon and mild in nature.

The ease of insertion was similar for both devices. However, when the study using laryngoscope guided insertion was excluded, the i-gel showed a higher incidence of insertion without resistance than the LMA-P. The large cuff of the LMA-P which can impede digital intra-oral positioning and propulsion into the pharynx, and the lack of a back-plate which makes the cuff more likely to fold over at the back of the mouth can cause difficulties in LMA-P insertion [8]. However, different authors reported “ease of insertion” in different ways, and the subjective nature of this assessment may have introduced a bias [24].

Insertion time was shorter with the i-gel than in with the LMA-P. Different studies allowed different numbers of attempts at successful insertion. Four studies allowed 2 insertions [12,13,15,22]; 3 studies allowed 3 attempts [4,10,11,14,23], and 4 of 9 studies did not specify the details of how insertion time was determined, which suggests that insertion times may not be directly comparable between studies. These potential methodological differences could be responsible for the heterogeneity associated with insertion time. The i-gel showed a tendency towards shorter insertion time compared to the LMA-P, which may be partly explained by the non-inflatable cuff in the i-gel requiring a shorter time to achieve an effective airway [25]. This may also explain the finding that the i-gel had a higher score for ease of insertion than the LMA-P.

The use of neuromuscular blocking drugs may affect the insertion time for airway devices. However, in this systemic review, the insertion time was no different between the 2 devices regardless of the use of neuromuscular blocking drugs. This finding might be explained by aspects of the LMA-P’s design that make it more difficult to insert than the i-gel, in particular the large cuff and lack of a back-plate.

OLP has been commonly used to assess successful airway placement in SGA studies. The leak pressure is an important indicator of both the success of positive pressure ventilation and the degree of airway protection [26]. The OLP of SGAs is determined by the strength of the seal between the surrounding soft tissue of the neck (including pharyngeal muscle) and the cuff of the mask. It has been reported that neuromuscular blockage relaxes the muscles of the neck, thus decreasing the OLP for the LMA-P [27]. In the present meta-analysis, the OLP was higher for the LMA-P than for the i-gel when a neuromuscular blocking drug was used. This may be due to the inflatable cuff of the LMA-P is better able to adapt to variations in pharyngeal anatomy than the non-inflatable cuff of the i-gel. This might be especially apparent in paralyzed patients [13].

This finding suggests that the LMA-P is a more effective ventilation device than the i-gel in paralyzed patients. This may be of particular relevance in patients with high airway pressure, such as obese patients undergoing intra-abdominal or laparoscopic surgery, patients undergoing lithotomy, patients undergoing surgery in the head-down position or patients with restrictive pulmonary disease [8].

The results of the meta-analysis did not indicate any significant difference in the quality of fiberoptic view between the i-gel and LMA-P. This suggests that these 2 devices can provide a similarly useful conduit in difficult airway management and failed intubation under general anesthesia. However, the number of studies included in the analysis was small and there was substantial heterogeneity. Therefore, the results should be interpreted with a degree of caution.

Complications arising from insertion of the 2 devices were minor and infrequent. Blood staining of the device (during anesthesia), sore throat and dysphagia (after anesthesia) were lower for the i-gel than for the LMA-P. These complications may be caused by the inflatable cuff of the LMA-P compressing microvascular structures and terminal nerve endings in these tissues [3]. In contrast, the i-gel has a non-inflatable cuff designed to provide an anatomical fit over these perilaryngeal structures [9].

The present work has several limitations. First, there are a number of potential sources of clinical and methodological heterogeneity between the evaluated studies including differences in the definition of insertion time, the use of neuromuscular blocking drugs, the induction, maintenance, and the depth of anesthesia, the patient population studied, and the expertise of the anesthesiologist inserting the device. Despite the fact that we conducted subgroup and sensitivity analyses to try to control for some of these factors, we could not account for all possible confounding factors when designing the study. Second, there is also the possibility of publication bias resulting from unpublished studies because of null results or small sample size. However, the present analysis did not seem to be hindered by publication bias, as assessed by Begg's funnel plot and Egger's test. Third, the analysis was based on a relatively included small number of studies. Further studies examining, a larger numbers of randomized controlled trials are required to confirm our findings. Fourth, because the sample size of the included studies was not large enough, cautious interpretation of rare events and safety analysis were necessary.

Regardless of limitations, our study applied rigorous methodology to compare the i-gel and the LMA-P, and was the first systematic review of this topic.

In conclusion, the i-gel was similar to the LMA-P with respect to the insertion success rate on first attempt, the quality of the fiberoptic view and the success rate of gastric tube insertion. Insertion time was shorter with the i-gel than with the LMA-P. The OLP for both devices was sufficiently high to protect the patient’s airway; however, the OLP in LMA-P was higher than when the i-gel was used in non-paralyzed patients. Complications experienced with the use of either device were minor and infrequent, but, the i-gel had advantages over the LMA-P in terms of lower incidences of blood staining, sore throat and dysphagia.

## Supporting Information

S1 Checklist PRISMA Checklist. (DOC)

S1 Appendix Search terms for MEDLINE and Embase. (DOC)
