# Supplementary material for: Comparison of the I-Gel and the Laryngeal Mask Airway Proseal during General Anesthesia: A Systematic Review and Meta-Analysis
Source: PLoS One. 2015 Mar 26;10(3):e0119469. doi: 10.1371/journal.pone.0119469 (PMC4374933; doi:10.1371/journal.pone.0119469)
Supplement: S1 Appendix — (DOC) [file pone.0119469.s002.doc]

**Search terms for MEDLINE**

1. randomized controlled trial.pt
2. randomized controlled trial$.mp
3. controlled clinical trial.pt
4. controlled clinical trial$.mp
5. random allocation.mp
6. exp double-blind method/
7. double-blind.mp
8. exp single-blind method/
9. single-blind.mp
10. or/1-9
11. clinical trial.pt
12. clinical trial$.mp
13. exp clinical trial/
14. (clin$ adj25 trial$).mp
15. ((singl$ or doubl$ or tripl$ or trebl$) adj25 (blind$ or mask$)).mp
16. random$.mp
17. exp research design/
18. research design.mp
19. or/11-18
20. 10 or 19
21. Case report.tw.
22. Letter.pt.
23. Historical article.pt.
24. Review.pt.
25. or/21-24
26. 20 not 25
27. exp laryngeal masks/
28. laryngeal mask airway.mp.
29. (LMA or ProSeal).mp.
30. (mask adj6 airway).mp.
31. or/27-30
32. i gel.mp.
33. igel.mp.
34. i-gel.mp.
35. or/32-34
36. 31 and 35
37. 26 and 36

**Search terms for Embase**

1. randomi?ed controlled trial$.mp.
2. 'controlled clinical trial (topic)'/exp
3. controlled AND clinical AND trials
4. controlled clinical trial$.mp.
5. 'randomization'/exp
6. 'random allocation'/exp
7. random allocation.mp.
8. double-blind.mp.
9. single-blind.mp.
10. #1 OR #2 OR #3 OR #4 OR #5 OR #6 OR #7 OR #8 OR #9
11. 'clinical trial (topic)'/exp
12. clinical AND trial$.mp.
13. random$.mp.
14. rct
15. #11 OR #12 OR #13 OR #14
16. #10 OR #15
17. 'case study'/exp
18. 'case report'/exp
19. 'abstract report'/exp
20. 'letter'/exp
21. #17 OR #18 OR #19 OR #20
22. #16 NOT #21
23. 'laryngeal mask'/exp
24. laryngeal mask airway.mp.
25. 'lma'/exp
26. Proseal
27. #23 OR #24 OR #25 OR #26
28. i AND 'gel'/exp
29. igel
30. ‘i-gel’
31. #28 OR #29 OR #30
32. 31 and 35
33. 26 and 36
